# Effects on Stroke Metrics and Outcomes of a Nurse-led Stroke Triage Team in Acute Stroke Management

**DOI:** 10.7759/cureus.5590

**Published:** 2019-09-07

**Authors:** Caleb J Heiberger, Stephanie Kazi, Tej I Mehta, Clayton Busch, Jessie Wolf, Divyajot Sandhu

**Affiliations:** 1 Radiology, University of South Dakota Sanford School of Medicine, Sioux Falls, USA; 2 Neurology, University of South Dakota Sanford School of Medicine, Sioux Falls, USA; 3 Neurology, Sanford Health, Sioux Falls, USA; 4 Interventional Neurology, Sanford Health, Sioux Falls, USA

**Keywords:** stroke, nurse, ischemic stroke, outcomes, emergency triage assessment and treatment

## Abstract

Background

Timely administration of healthcare in acute stroke, congruent with national stroke metrics, relates to better patient outcomes. A nurse-led stroke triage team instituted at our facility was hypothesized to improve metrics and outcomes. To evaluate the effect of the nurse-led stroke triage team we compared specific stroke metrics and patient outcomes before and after the program initiation.

Methods

In retrospective review, we analyzed stroke metrics one year prior to the start of the triage program (controls) and one year after the start of the program (cases), including the following metrics: patient arrival, emergency department assessment, neurology contact, head computed tomography (CT) scan, and delivery of tissue plasminogen activator (tPA) or puncture for mechanical thrombectomy. Primary outcome measures were improved metric times.

Results

Ninety-five acute stroke events were analyzed: 26 controls and 69 cases. Cohort demographics included means of age 72.82 years, National Institutes of Health Stroke Scale (NIHSS) 15.96, discharge and 90-day mRS 3.71 and 3.55 respectively, and length of stay 5.98 days. There were significantly different improvements in metrics between arrival time to CT start, emergency room physician evaluation to CT start, neurology contact to CT start, and neurology contact to tPA initiation for cases post-triage team institution. No significant differences during this period were seen for other metrics. Multivariate analysis controlling for age, sex and NIHSS found no significant difference for discharge or 90-day mRS scores.

Conclusions

An interdisciplinary approach to acute stroke management can impact stroke metrics. These data support the integration of specially trained stroke nurses in acute stroke triage for quality improvement efforts.

## Introduction

Stroke is the fifth leading cause of death in the United States. Despite major advancements in recent years, it remains a significant cause of morbidity and mortality worldwide with an associated financial burden [1]. The American Heart Association/American Stroke Association (AHA/ASA) has proposed metrics to monitor the quality of care provided at stroke centers and to facilitate quality improvements at individual centers [2]. Fulfillment of stroke metrics (e.g. admission National Institutes of Health Stroke Scale [NIHSS], door-to-needle, door-to-CT times) has been positively associated with improved patient-centered outcomes [3]. Owing to the time-sensitive nature of acute stroke care, efficient, enhanced emergent care is a key priority. Metrics have improved significantly in recent years, but modifiable external factors remain open to further improvement. Management of acute stroke by different health providers, teams, and units has been shown to impact specific stroke metrics [4, 5]. Specifically, the Stroke Fast Track program identified that nurse case management of acute stroke significantly improves triage-to-treatment time with intravenous tissue plasminogen activator (tPA) concomitant with improved NIHSS scores at 24 hours post-treatment [6]. Other studies have found similar results and identified that registered nurses (RN's) trained in acute stroke triage significantly improve adherence to stroke management guidelines [7]. To better elucidate the effects of a nurse-led triage program on acute stroke metric times and patient outcomes, we established such a program at our institution that places nursing staff as the primary first contact in acute stroke management. 

The goal of the redesign was to improve time to stroke metrics by maximizing the efficiency of stroke patient triage. The main difference in operations between periods before and after the program involved delegating certain stroke code responsibilities previously managed by the emergency department (ED) physicians to stroke trained RN's. Prior to the program, the ED physician was responsible for immediate care of the patient upon arrival and ongoing management, as well as reporting NIHSS, last known well or onset of symptoms, and initiating contact with neurology. Following program initiation, RN’s trained in ischemic stroke management (e.g. treatment protocol, rapid triage, and NIHSS), were designated as the stroke RN and assumed some of the ED physicians’ responsibilities. The stroke RN was tasked to assist in patient arrival and direct admission to CT, remaining with them throughout the entire duration of their assessment to obtain a patient report. They streamlined pertinent stroke history (last known well, the onset of symptoms, NIHSS score, large vessel occlusion assessment, tPA contraindications) while putting the patient on a CT table, synthesized information for the direct report to the neurologist, and obtained a plan of care pending CT results. A goal was set to report the findings to the covering neurologist within 15 minutes of the patient’s arrival. Meanwhile, the ED physician completed a further assessment and the ED RN (not the same as designated stroke RN) initiated intravenous (IV) lines and rapid lab collection. During both pre- and post-program initiation an in-house neurologist was present from 08:01-17:00 and an on-call neurologist was available from 17:01-08:00. 

## Materials and methods

Study design

In retrospective review, we analyzed stroke metrics one year prior to the start of the triage program (controls) and one year after the start of the program (cases), including the following metrics: patient arrival, emergency department assessment, neurology contact (time of ED physician or stroke RN report to neurologist), head CT scan, and delivery of tPA or puncture for mechanical thrombectomy. 

Inclusion/exclusion criteria 

All acute ischemic stroke patients treated with intravenous tPA or mechanical thrombectomy at our institution between 01/01/17 and 03/01/19 were included in this study. The nurse-led triage program was instituted on 02/01/18. Acute ischemic stroke events treated prior to this date were included in the control group, and events after this date were included in the case group. 

Data collection

The following metrics were collected relative to patient arrival time: time of emergency department assessment, neurology contact, head CT scan, and delivery of tPA or puncture for mechanical thrombectomy. Primary outcome measures included improved metric times relative to patient arrival. Secondary outcomes included improved metric times relative to other recorded significant events (i.e. time from head CT scan to delivery of tPA), modified Rankin Scale (mRS) score at discharge, mRS score at three-month follow-up, and length of hospital stay. Demographic data including age, gender, race, and arrival NIHSS scores were also collected.

Data analysis

Statistical analysis was performed using R, Software Environment for Statistical Computing Version 3.5.3. For univariate analyses, differences in continuous or ordinal mean scores of demographic data and explanatory measures between the dichotomous summary measures of in-house vs. no in-house neurologist hours (between 08:01 -17:00 and between 17:01 - 08:00 respectively) and pre- vs. post-stroke nurse triage program institution were examined using independent t-tests for normally distributed data and the Mann-Whitney U test for skewed data. Multivariate models adjusted for age, gender and NIHSS scores were generated to determine the significance and effect size of the stroke nurse triage program on primary and secondary outcomes. Kaplan-Meir survival curves were generated to graphically represent 90-day mortality post-stroke. Log-rank tests, unadjusted and adjusted Cox proportional hazard ratios were generated to determine the significance of the stroke nurse triage program on post-stroke survival.

## Results

Over the two years of data collection, there were 95 patients with acute ischemic stroke eligible for inclusion in the study. The mean age of the entire cohort was 72.82 years. There was an approximately equal sex distribution, a mean NIHSS of 15.96, mean mRS score at the discharge of 3.71, mean mRS at 90 days post-discharge of 3.55 and mean length of stay of 5.98 days. Of the two cohorts, there were 26 control patients and 69 experimental cases. There was a significant difference in baseline mean NIHSS scores between the two groups (control: 13.15, cases: 17.04; p=0.041). Complete demographics and results for functional outcomes pre- and post-program are listed in Table 1.

**Table 1:** Patient characteristics and study outcomes NIHSS - National Institute of Health Stroke Scale; mRS - modified Rankin Scale; ED - emergency department; SD - standard deviation; CT - computed tomography; tPA - tissue plasminogen activator Outcomes are unadjusted and contain data from all hours

Variable	Controls (n=26)	Cases (n=69)	p-diff
Mean age (SD)	74.12 (15.37)	72.33 (13.74)	0.587
Female (%)	14 (53.8)	32 (46.4)	0.675
Caucasian (%)	26 (100.0)	63 (91.3)	0.299
Hispanic (%)	0 (0.0)	1 (1.4)	
Native American (%)	0 (0.0)	5 (7.2)	
Mean NIHSS (SD)	13.15 (8.93)	17.04 (7.81)	0.041
Mean discharge mRS (SD)	3.42 (1.60)	3.81 (1.51)	0.244
Mean 90 Day mRS (SD)	3.18 (2.40)	3.73 (2.22)	0.356
Mean length of stay (SD)	5.92 (5.99)	6.00 (5.00)	0.950
Arrival to ED assessment (SD)	4.50 (5.71)	2.84 (3.75)	0.102
Arrival to neurology contact (SD)	6.65 (8.65)	5.96 (10.02)	0.116
Arrival to CT start (SD)	17.08 (13.70)	8.52 (10.02)	0.001
Arrival to tPA start (SD)	67.62 (60.80)	48.11 (27.33)	0.227
Arrival to groin puncture (SD)	78.50 (41.73)	66.80 (30.34)	0.233
ED assessment to neurology contact (SD)	2.15 (7.90)	3.12 (10.58)	0.675
ED assessment to CT (SD)	12.64 (11.94)	5.68 (10.73)	0.008
ED assessment to tPA (SD)	61.50 (58.03)	45.28 (29.00)	0.302
ED assessment to groin puncture (SD)	76.50 (42.44)	63.88 (30.28)	0.200
Neurology contact to CT (SD)	10.16 (8.82)	2.57 (9.76)	0.001
Neurology contact to tPA (SD)	57.94 (55.15)	33.11 (24.68)	0.050
Neurology contact to groin puncture (SD)	76.07 (41.54)	62.66 (29.19)	0.161

Primary outcomes

Pre- vs. post-program - all hours

For non-stratified analysis, there were significant differences in time measures (minutes) between arrival time to CT start (control group: 17.8, case group 8.52; p=0.001) , emergency room physician evaluation to CT start (control group: 12.64, case group: 5.68; p=0.008), neurology contact to CT start (control group: 10.16, case group: 2.57; p=0.001), and neurology contact to tPA (control group: 57.94, case group: 33.11; p=0.05). No significant differences were noted during this period for other metrics including time from arrival to neurology contact, emergency department physician assessment, tPA start, groin puncture, nor the length of patient stay. However, door to both tPA start and groin puncture times were reduced and had smaller standard deviations amongst cases. Complete results for stroke metrics time pre and post-program are listed in Table 1. 

Pre- vs. post-program - “on” hours

Among acute stroke cases presenting during “on” hours (when an in-house neurologist was present),, there was a significant difference in time to emergency room physician assessment from patient arrival (control group: 5.71, case group: 2.58, p=0.047). No significant differences were noted for other metrics during this time. The group sample sizes with this stratification were 14 and 33 for the control and case groups respectively. 

*Pre- vs. post-program - “off” hours* 

Among acute stroke cases presenting during “off” hours (when an in-house neurologist was not present), there were significant differences in time measures between arrival time to CT start (control group: 18.33, case group 8.47; p=0.011), emergency room physician evaluation to CT start (control group: 15.25, case group: 5.39; p=0.014) and neurology contact to CT start (control group: 11.75, case group: 1.17; p=0.002). The group sample sizes with this stratification were 12 and 36 for the control and case groups respectively. 

Multivariate analyses

For non-stratified and stratified data, all primary outcomes found to be significant in unadjusted analysis retained significance in multivariate analysis when adjusted for age, gender and NIHSS. 

Secondary outcomes

**Figure 1:**
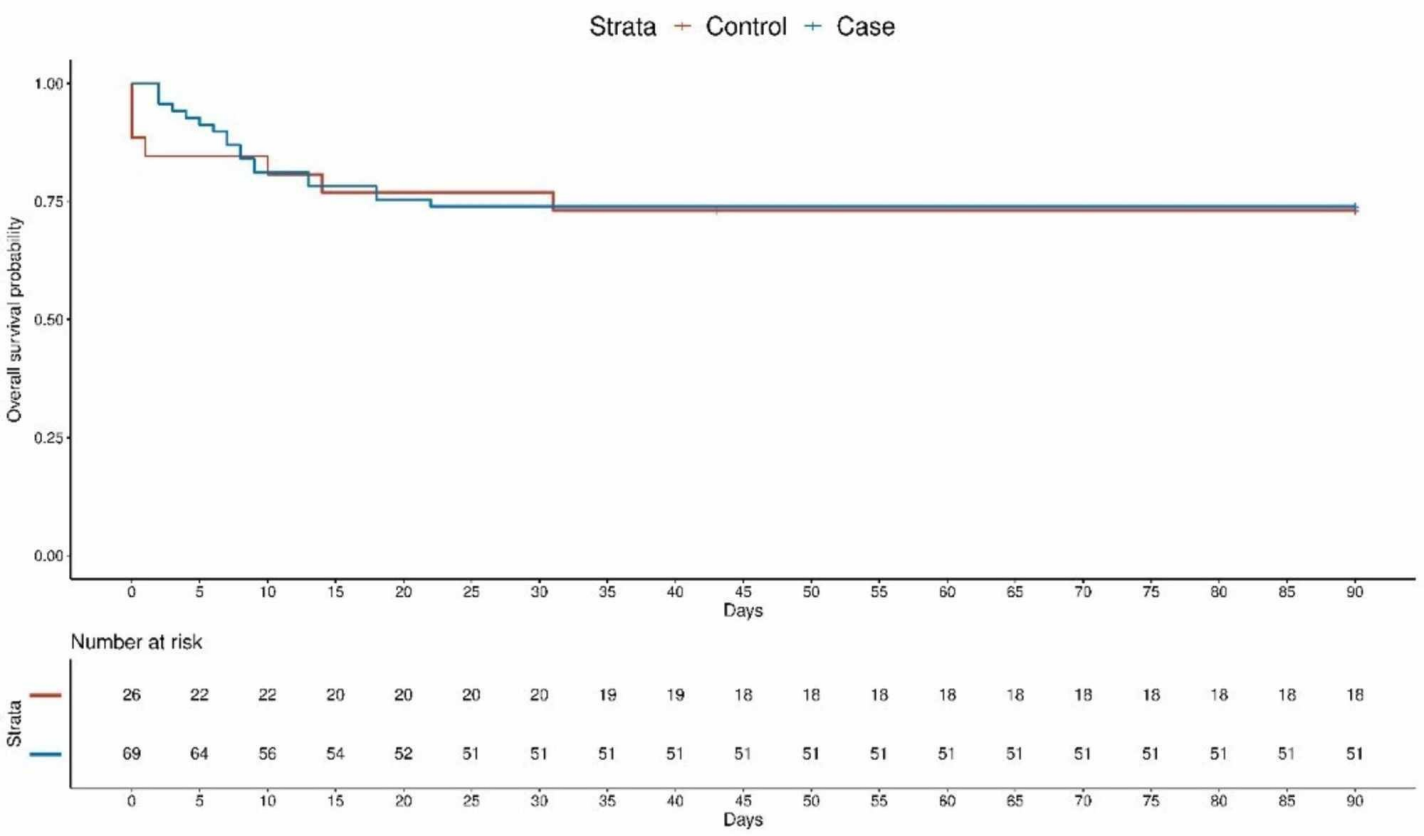
Ninety-day mortality Kaplan-Meir survival plot

Neither unadjusted nor adjusted analyses identified significant differences in discharge or 90-day mRS outcomes between cases and controls. There were no significant differences in 90-day survival post-stroke between cases and controls nor between patients in on- vs. off-hours (log-rank test, p = 0.9 and 0.5 respectively). Figure 1 illustrates the 90-day Kaplan-Meir survival plot for controls and cases in this study. The median survival time was not reached: 69% of controls and 74% of cases survived to 90-days post-stroke. 

Unadjusted Cox proportional hazard ratios did not identify a significant survival effect after the institution of the stroke nurse triage program (coefficient = 0.9287, 95% confidence interval [CI] 0.3878 - 2.224). Cox proportional hazard model adjusted for NIHSS scores, age, sex and including an interaction variable for “on” vs. “off” hours pre- and post-program institution did not identify significance of any of the aforementioned variables (coefficient off-hours = 0.6996, 95% CI 0.1517 - 3.226, coefficient cases = 0.6654, 95% CI 0.1978 - 2.238, coefficient interaction = 1.2081, 95% CI 0.1942 - 7.514). 

## Discussion

Implementation of the nurse-led stroke team improved time sensitive metrics of stroke care and increased institutional compliance with recommended national guidelines. The AHA/ASA guidelines for acute ischemic stroke recommend that a head CT be obtained within 20 minutes of patient arrival and IV-tPA be initiated within 60 minutes of patient arrival [8]. Prior to initiation of the program, the average time to head CT was within the recommended window (17.08 minutes), but stroke nurse-led cases demonstrated significant improvement with the average time to CT of 8.52 minutes. The stroke nurse-led program improved door-to-needle times from 67.62 minutes to 48.11 minutes. Although the time difference was non-significant (p=0.227), the improvement reached compliance with AHA/ASA guidelines whereas the previous time did not. With fewer than 30% of primary stroke centers meeting criteria for less than 60-minute door-to-needle times [9], nurse-led stroke team implementation may be an effective method of improvement. 

The improved metric times following onset of our program were not associated with better outcomes at discharge or 90 days, per mRS scores. A recent Australian study by Middleton et al. evaluated the effects of targeting nursing in acute stroke triage, treatment, and transfer, in which significance was not found in any of their metrics regarding clinician behavior or stroke outcomes [10]. The 90-day mRS outcomes of our study were comparative to Middleton’s outcomes, however, we did find differences in clinician behavior, although using different metrics (adherence to timing). 

It has been established that faster times to treatment with tPA improve patient outcomes [11-14]. Despite this, the shortened time to tPA delivery during this study was not associated with better outcomes. Limited evidence is available to indicate if achieving door-to-needle times under 60 or even 90 minutes has a consistent impact on functional outcomes, especially on mRS [15]. Therefore, while our mean door-to-needle time decreased by nearly 20 minutes, it remains unclear if such a change should significantly improve outcomes. 

Additionally, the significantly higher mean admission NIHSS score in the nurse-run cases presents as a confounding variable in two distinct ways. First, being that large NIHSS values are predictive of worse functional outcomes, possibly explaining the lack of significant improvement following the program’s initiation [16]. Second, the acuity of patients with higher NIHSS values has been indicated to drive shorter door-to-needle times [17]. This could explain, in part, the variance in door-to-needle times between the two groups, although the concomitant improvement of other metrics in the study makes NIHSS score variance unlikely to be the entirety driving reason behind the difference. Further, multivariant analysis of metric times and mRS outcomes controlling for age, sex, and NIHSS values demonstrated the persistence of the significant difference or lack thereof between cases and controls for metric times and mRS outcomes, respectively. Therefore, it may be concluded that placing specially trained nurses as leaders in the stroke triage process can improve quality of care for patients as indicated by improved metric times. These findings support other studies evaluating nursing management in acute stroke settings with regard to time-sensitive metrics [6, 18]. 

The small sample size limits the statistical power of this study. The availability of mortality outcomes was limited by the follow-up period not extending beyond 90 days for many of the patients in the post-program cohort. Variances in the program’s design compared to other interdisciplinary models limit the generalizability of the study results. Future works may focus on expanding sample sizes and follow-up length as well as exploring other measures of functional outcomes aside from mRS scores and mortality. 

## Conclusions

Interdisciplinary care of patients suffering acute stroke remains an area for improving stroke management. The program described herein used a synergistic collaboration of nursing and physician-led care to improve time-sensitive stroke metrics and adherence to national guidelines. These data support the integration of specially trained stroke nurses in acute stroke triage to improve stroke metrics and facilitate quality improvement. 
